# Effects of processing on the polyphenolic content of Armagh Bramley apple products and pomace

**DOI:** 10.1038/s41598-025-31695-7

**Published:** 2025-12-18

**Authors:** Ruth Loy, William C. McRoberts, Alan Gordon, Chris I. R. Gill, L. Kirsty Pourshahidi

**Affiliations:** 1 Agri-Environment Branch, Environment and Marine Sciences Division, Agri-Food and Biosciences Institute, Newforge Lane, Belfast, BT9 5PX UK; 2 Nutrition Innovation Centre for Food & Health (NICHE), Ulster University, Coleraine, Northern Ireland BT52 1SA UK

**Keywords:** Armagh Bramley, Apple, (Poly)phenol, Ascorbic acid, Antioxidant, Processing, Analytical biochemistry, Mass spectrometry, Secondary metabolism

## Abstract

The content of health promoting (poly)phenols, ascorbic acid and antioxidants is currently unknown in products produced from the Armagh Bramley apple. Four industrially processed products (diced apple, puree, canned sliced apple and apple sauce), one artisan product (apple butter) and two newly developed products (apple sauce and apple butter) were compared against raw material, with waste pomace also assessed. (Poly)phenol and ascorbic acid content were quantified by LC-UV-DAD. (Poly)phenol content of apple products ranged from 726 to 1919 mg kg^–1^ FW. Significant differences were observed between (poly)phenol content of products, demonstrating that (poly)phenolic profile and content is impacted by processing method. The highest mean (poly)phenol content was observed in industrially produced apple sauce and the lowest in an artisan apple butter product which also had the highest antioxidant capacity. Ascorbic acid was reduced by up to 97% by processing. (Poly)phenolic compounds were present at significant levels worthy of valorisation in waste after processing for all products ranging from 1974 to 5588 mg kg^–1^ FW. The data clearly shows that processing affects the (poly)phenol content and profile of Armagh Bramley apple products and consequently optimisation of processing technique has the potential to preserve greater bioactive content.

**Supplementary Information:**

The online version contains supplementary material available at 10.1038/s41598-025-31695-7.

## Introduction

The World Health Organisation recommends at least 400 g of fruit and vegetables per day^1^, with apples considered to be one of the world’s most commonly consumed fruits^2^. Studies have shown that (poly)phenols are the dominant antioxidant species in apple^3^. Apples typically contain 5.3–27.2 g kg^–1^ DW of (poly)phenols^4^, with apples demonstrating a protective effect from variety of chronic diseases^5–9^. Apples are also a rich source of dietary fibre as well as other antioxidants such as ascorbic acid^10^.

Northern Ireland (NI) produces a unique apple cultivar with an EU Protected Geographical Indication (PGI) status^11^ known as the ‘Armagh Bramley’ which comprises 99% of the 57,300 tonnes of apples grown per annum in this region^12^. These apples are NI’s largest fruit commodity with a market value of £16.4 million, historically supplying over 60% of the UK culinary market^12,13^. A study which compared apples by group reported that the culinary group which included Bramley’s seedling and Armagh Bramley apples were significantly higher in (poly)phenols than dessert apples, containing 16.4 g kg^–1^ DW^14^. The Armagh Bramley can be processed by a variety of techniques, including both artisan and industrial processes, with up to 84% processed into products such as juice, cider, puree, pie filling and sauce (DAERA, personal communication, 2021). Studies investigating the effects of processing on (poly)phenolic content in apple products are limited, however, it has been suggested (poly)phenolic composition of apple sauce is similar to apple flesh^15^. Furthermore, products which retain whole apple, may retain higher (poly)phenol content than those produced from apple juice, which has been associated with a loss in phenolics^16,17^. Thus, it could be hypothesised that the processing technique(s) will inform the resultant bioactive content of Armagh Bramley processed products.

Waste from apple processing known as pomace was shown to contain the same classes of (poly)phenols in comparable quantities to unprocessed apple and measured in the 5 g kg^–1^ DW range^18^. Representing around 25% of the whole apple, pomace is comprised of 95% skin and flesh, 2–4% seeds and 1% stems^19^. By estimation, NI could potentially produce up to 14,000 tonnes of pomace per year, which can have a variety of further uses such as dietary supplements as well as the extension of shelf life of meat through inclusion in active packaging and edible films^20–22^. Efficient and innovative use of by-products from the apple industry could have an important role to play in maximizing value from NI food production and is an important component of ‘circular’ economy development.

The aim of this study was to assess and compare the (poly)phenolic and antioxidant content of Armagh Bramley products and pomace.

## Materials and methods

### Sampling: initial screening

An initial screening study was conducted to determine the (poly)phenolic and antioxidant content of apple products produced or available in NI. Commercially available jams, jellies, sauces and purees (total n = 13) were purchased from artisan outlets and supermarkets in 2019. Of the thirteen products screened three specifically included “Armagh Bramley” as an ingredient, while a further four stated “Bramley” on the packaging. A subsample from three replicate jars of each product was subjected to the following analyses: dry matter content; Brix; pH; and three antioxidant assays (total (poly)phenols, ABTS, DPPH).

The results from the initial investigation were used to select products for a targeted study. Lower sugar content/lower pH coupled with higher estimation of (poly)phenols and antioxidant capacity in industrially produced apple sauces informed the decision to include similar pulp containing products. Chutneys and preserves were not included due to higher sugar content and presence of other fruit. Industrial producers in NI mainly produce intermediate products which are exported to the UK and Republic of Ireland (ROI) (MacNiece Fruit personal communication, 2024) for further processing, therefore four common Armagh Bramley products containing apple pulp were selected. Apple butter was also selected despite its high sugar content as this product had the highest estimation of (poly)phenol content and antioxidant capacity (ABTS). Two new products (apple sauce and apple butter) were developed using common recipes (NPD) to mimic small scale artisan production.

### Sampling: targeted study

**Fig. 1:**
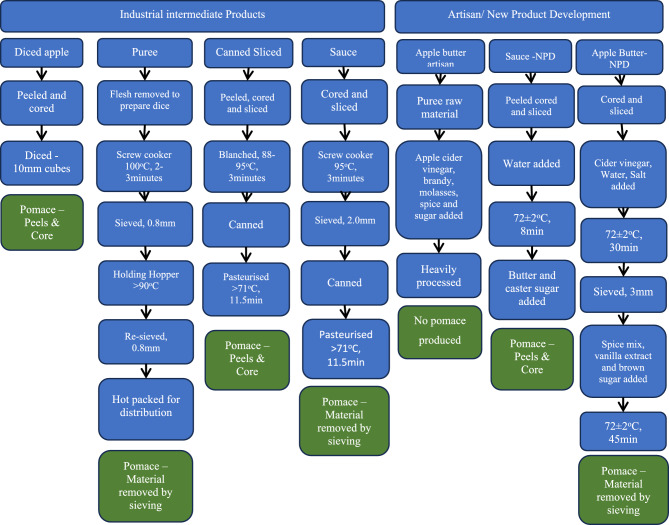
Processing flow for each product. Producers of apple butter—artisan declined to include full processing information due to commercial sensitivities. Industrial sauce and apple butter NPD pomace do not include core which was discarded early in the production process.

Four different industrial products originating from two separate processing plants in NI were each collected on three occasions. Diced apple and apple puree, both products were frozen immediately after production and stored in polythene bags (Davidsons Quality Foods, 20 Cannagola Beg Road, Portadown, Co. Armagh, BT62 1RR, N.I) and were collected during 2020–2021. Apple sauce and canned sliced apple, both products were received canned (MacNiece Fruit LTD, 65 Ardress Road, Portadown, Co Armagh, BT62 1SQ, NI) and were collected during 2020–2021. One apple butter artisan product was collected from 2020–2021. Two new products (apple sauce and apple butter) were developed (NPD) in the AFBI sensory suite (AFBI Headquarters, Belfast; apples were donated by Glass Brothers, 132 Killmore road, Co. Armagh, BT61 8NR, NI) in 2022, these products were frozen immediately after production. Each collection included apple (raw material) consisting of 12 apples from the batch used to prepare products (500 g puree for apple butter—artisan), as well as approximately 500 g pomace frozen on the day of production by the producer (not produced for apple butter—artisan). Process flow for each product is described in Fig. 1, with full details for each product included in supplementary material.

### Sample preparation

All samples were processed in the laboratory on the day of production. In the case of the representative raw material, 12 apples were randomly divided into 3 replicates each consisting of 4 apples. These four apples were then quartered and one segment from each combined for use in subsequent experimental analysis; the remaining segments discarded. Segments were then sliced finely and immediately frozen in liquid nitrogen before being lyophilised using a Christ Beta 1–8 LSC plus lyophiliser (Sciquip, Newtown, UK), to preserve metabolites^23^. For apple butter—artisan (puree) raw material, intermediate/artisan products and pomace, 150 g replicate portions were retained from each batch for analysis. Samples if not already, were frozen before being lyophilised. Lyophilised samples were milled using a coffee grinder and stored at – 20 °C prior to analysis. Sample weights (g) taken throughout this process were used to calculate dry matter (%).

### Standards and reagents

Analytical grade solvents were used unless specified otherwise, (purity = 95–99.5%). Folin & Ciocalteu’s reagent (2 M), sodium carbonate, 2,2-diphenyl-1-picrylhydrazyl (DPPH), 2,2’-azinobis(3-ethylbenzothiazoline-6-sulfonic acid (ABTS), gallic acid, acetic acid, (±)−6-hydroxy-2,5,7,8-tetramethylchromane-2-carboxylic acid (TROLOX), quercetin 3-glucoside, p-coumaric acid, chlorogenic acid, phloridzin dihydrate, phloroglucinol, ascorbic acid, sodium acetate, formic acid, monopotassium phosphate and potassium persulfate were purchased from Sigma (Steinheim, Germany). The standards (+)-catechin, (-)-epicatechin, procyanidin B1, procyanidin B2, quercetin 3-O-rutinoside, quercetin 3-O-galactoside and cyanidin 3-O-galactoside chloride were purchased from Extrasynthese (Lyon, France). Metaphosphoric acid, acetonitrile (HPLC grade), hydrochloric acid and ethanol were obtained from Fisher Scientific (Swindon, United Kingdom) with methanol (Romil SpS, super purity solvent, H410), obtained from Analab (Lisburn, United Kingdom).

### Soluble solids and pH

Soluble solid content was measured with a portable refractometer (RHB-90ATC., Labtek services, UK). One drop of product was added to the refractometer prism and SCC read as Brix^o^. pH was measured using a pH meter (Orion Start A111 benchtop pH meter with refillable 9157BNMD triode (Thermo scientific.,Paisley, UK) calibrated prior to use with phosphate buffers at pH4 and pH7.

### Extraction of unbound (poly)phenolic compounds

Unbound (poly)phenols were extracted for the determination of (poly)phenolic content and antioxidant capacity according to a modified method^24–26^. In brief 500 ± 0.5 mg of lyophilised apple powder was initially homogenised using a T-25 Ultra-Turrax (IKA Labortechnik, Stauden, Germany), (30 s) with 5 ml of 80% aqueous acidified methanol (1% acetic acid) and allowed to stand at room temperature (1 h) before centrifugation (10 min, 3849 g, + 4 °C). The supernatant was collected and the remaining (poly)phenols extracted from the residue with a further 5 ml of 80% aqueous acidified methanol (1% acetic acid). The supernatants were combined and made up to 10 ml with 80% aqueous acidified methanol (1% acetic acid) and subsequently filtered through a syringe filter (Whatman puradisc 13 mm, 1.0 µm PTFE). Extracts were stored at – 20 °C pending analysis.

### (Poly)phenol analysis

Thirteen of the most common (poly)phenol compounds found in apples were quantified by HPLC UV-DAD^27^ including catechin, epicatechin, procyanidin B1 and B2, chlorogenic acid, p-coumaroylquinic acid, phloridzin, phloretin xyloglucoside, quercetin 3-rutinoside, quercetin 3-galactoside, quercetin 3-glucoside, quercetin 3-rhamnoside and cyanidin 3-galactoside. Analyses were performed on an Agilent 1200 series HPLC system coupled to an Agilent 6510 QTOF mass spectrometer (Agilent Technologies., Aldbronn, Germany) equiped with a diode array detector prior to the mass spectrometer. Separation was achieved using a Phenomenex Luna 5 µm C18(2) 100 Å LC (150 mm × 2.0 mm) column maintained at 30 ⁰C. The mobile phase consisted of deionised water containing 1% formic acid (mobile phase A) and 100% acetonitrile containing 1% formic acid (mobile phase B). A gradient program was employed as follows: 0% B to 15% B in 45 min, 15% B to 30% B in 15 min, 30% B to 50% B in 5 min, 50% B to 100% B in 5 min. These conditions were held at 100% B for 3 min, then returned to starting conditions over 1 min and allowed to equilibrate for a further 6 min. The flow rate was held at 0.2 ml min^−1^ and 5 µl of each sample was injected. Simultaneous monitoring of UV signals at 280 nm (flavan-3-ol monomers, procyanidins and dihydrochalcones), 320 nm (phenolic acids), 360 nm (flavonols) and 530 nm (anthocyanins) was carried out. Where possible, compounds were quantified using calibration curves prepared with authentic standards. Where authentic standards were not available surrogates were utilised as described in previous studies, p-coumaroylquinic acid was quantified against p-coumaric acid and phloretin-2-xyloglucoside against phloridzin^25,28,29^. Recoveries, LOD and LOQ data derived during validation are provided in supplementary material (Table S1). Individual compounds have not been corrected for recovery. The total content of (poly)phenols was calculated by summing the individual (poly)phenolic compounds in each structural class. The conditions used to carry out qualitative confirmation using the QTOF electrospray interface were a gas temperature of 300 °C and flow of 11 L helium min^−1^. The nebuliser was set at 60 psi, capillary voltage at 4000 V and fragmentor at 100 V. Accurate mass data was collected in negative electrospray mode over a mass range of 100–1000 Da at a rate of 1.5 spectra s^−1^. Qualitive confirmation was not available for NPD products.

### Proanthocyanidin analysis

Proanthocyanidins were quantified following a modified acid-catalysed depolymerisation in the presence of phloroglucinol, resulting in a mixture of flavan-3-ol monomers composed of existing monomers and terminal subunits along with phloroglucinol adducts which correspond to extension subunits. The quantity of these analytes was determined by HPLC–UV-DAD^30^. Briefly, 800 µl of a 0.1 N HCl/methanol solution containing, 50 g L^−1^ phloroglucinol and 10 g L^−1^ ascorbic acid was added to 50 ± 1 mg of freeze-dried sample and homogenised using a laboratory vortex mixer. The sample was incubated (20 min, 50 °C) before immediately cooling in an ice bath (5 min). Sodium acetate (1 ml, 40 mM) was added, and the sample was centrifuged (1946 g, 10 min, + 4 °C). The resulting supernatant was used for HPLC analysis. Quantification was performed on an Agilent 1260 series HPLC system (Agilent Technologies., Aldbronn, Germany) equipped with a diode array detector (DAD). Separation was achieved using a Phenomenex Luna 5 µm C18(2) 100 Å LC (150 mm × 2.0 mm) column operated at 30 °C. The mobile phase consisted of deionised water containing 1% formic acid (mobile phase A) and 100% acetonitrile containing 1% formic acid (mobile phase B). A gradient program was employed as follows: 0% B to 15% B in 45 min, 15% B to 100% B over 2 min, then held at 100% B for 5 min, returning to starting conditions over 1 min and allowed to equilibrate for a further 8 min. The flow rate was held at 0.2 ml min^−1^ and 5 µl of each sample was injected. The UV signal was continuously monitored at 280 nm. Existing monomers and terminal subunits of (+)-catechin and (–)-epicatechin were quantified using calibration curves prepared with authentic standards, with the phloroglucinol adducts quantified using an (-)-epicatechin calibration curve. Values for existing (+)-catechin and (–)-epicatechin monomers determined during initial (poly)phenol analysis were deducted prior to calculation of the degree of polymerisation. This method was validated by comparison to a method using thiolysis to depolymerise the procyanidin and this data with the LOD and LOQ data are provided in supplementary material (Table S2).

### Ascorbic acid content

Ascorbic acid was quantified using a previously described method^31^. In brief, 700 µl of a 3% metaphosphoric acid (w/v)/8% (v/v) acetic acid solution was added to 50 ± 1 mg of freeze dried sample. A further 300 µl of methanol was added and the solution, homogenised (1 min) using a laboratory vortex mixer prior to centrifugation (3713 g, 10 min, + 4 °C). The supernatant was subsequently filtered through a syringe filter (Whatman 13 mm, 0.45 µm PTFE). Quantification was performed on an Agilent 1200 series HPLC system (Agilent Technologies., Aldbronn, Germany) equiped with a diode array detector (DAD). Separation was achieved using a Phenomenex Synergi hydro-RP 4 µm 80 Å LC column (150 mm × 2.0 mm) column operated at 25 °C. The mobile phase consisted of aqueous monopotassium phosphate, KH_2_PO_4_ (5 mM, pH 4.8; mobile phase A) and 100% methanol (mobile phase B). A gradient program was employed as follows: 0% B held for 2 min, 0% B to 20% B in 3.5 min, returned to starting conditions over 2 min and allowed to equilibrate for a further 5 min. The flow rate was held at 0.2 ml min^–1^ and 1 µl of each sample was injected. A UV signal was continuously monitored at 260 nm. Ascorbic acid was quantified using a calibration curve prepared with an authentic standard. Recoveries, LOD and LOQ data derived during validation are provided in supplementary material (Table S3). Reported concentrations have not been corrected for recovery.

### Total (poly)phenols by Folin–Ciocalteu assay

The estimation of (poly)phenol content was carried out using the Folin & Ciocalteu total phenols spectrophotometric assay as described by Bobo-Garcia and colleagues (2015). Methanolic apple extracts (20 µl) and 100 µl of 2 M Folin & Ciocalteu’s reagent (pre diluted 1:4 in deionised H_2_0) were added to a 96 well plate and shaken for 4 min (600 rpm), then 75 µl of 100 g L^–1^ sodium carbonate solution was added and the plate shaken for 1 min (600 rpm) followed by a 2-h incubation (RT, in dark). Absorbance was determined using a Tecan spark multimode microplate reader (Tecan group LTD., Männedorf, Switzerland) equipped with SparkControl software version 3.0. The plate was shaken for 5 s at 1 mm amplitude before absorbances were measured at 750 nm using 25 flashes per well. Results were expressed as an average of triplicate measures against a Gallic acid standard curve as mg gallic acid equivalents GAE kg^–1^ FW.

### Antioxidant capacity: DPPH

The estimation of antioxidant capacity by DPPH (2,2-diphenyl-1-picrylhydrazyl) electron transfer assay was carried out using a modified method^32^. Methanolic apple extracts (20 µl) and 280 µl of DPPH radical solution (150 µmol L^–1^) were added individually to a 96 well plate and shaken for 45 min (350 ± 50 rpm). Absorbance was determined using a Tecan spark multimode microplate reader (Tecan group LTD., Männedorf, Switzerland) equipped with SparkControl software version 3.0 at 515 nm using 25 flashes per well. Results were expressed as an average of triplicate measures of TROLOX equivalents against a TROLOX standard curve as mmol TROLOX eq kg^–1^ FW.

### Antioxidant capacity: ABTS

The estimation of antioxidant capacity by ABTS (2,2'-azino-bis(3-ethylbenzothiazoline-6-sulfonic acid) electron transfer assay was carried out using a modified method^33^. An ABTS ^•+^ radical cation solution was prepared in advance by adding an ABTS solution (7 mmol L^–1^, deionised water) to potassium persulfate solution (2.45 mmol L^−1^, deionised water) followed by a 16 h incubation (RT, in dark). Methanolic apple extracts (20 µl) and 280 µl of ABTS ^•+^ radical cation solution were added individually to a 96 well plate and the plate shaken for 4 min (600 rpm) followed by 30 min incubation (28 °C, in dark). Absorbance was determined using a Tecan spark multimode microplate reader (Tecan group LTD., Männedorf, Switzerland) equipped with SparkControl software version 3.0, at 734 nm using 25 flashes per well. Results were expressed as an average of triplicate measures of TROLOX equivalents against a TROLOX standard curve as mmol TROLOX eq kg^–1^ FW.

### Statistical analysis

Analysis of variance (one-way ANOVA) was carried out using GENSTAT (Windows 23rd edition, VSN International, Hemel Hemstead, UK). Four separate analyses were carried out, A) 13 products from the screening study (n = 3 replicates) were compared. B) Raw materials from 7 (n = 3 replicate batches) were compared (both NPD products were produced from the same raw material). C) Seven products analysed in this study (n = 3 replicate batches) and D) waste pomace (n = 3 replicate batches) from each of the 7 products investigated was compared (no waste material produced for apple butter—artisan). Where the overall treatment effect was significant then pairwise differences between the individual levels of the treatment were assessed using Fisher’s least significant difference test. Significant differences were defined as *p* < 0.05 thoughout. Data are presented in tabular form as a mean value ± standard deviation.

## Results

### Initial screening

Dry matter, ^o^Brix, pH and antioxidant capacity by three assays (TP, DPPH, ABTS) for 13 apple products subjected to initial screening are shown in Table 1. Statistical differences were observed for all metrics measured (*p* < 0.001). Apple butter manufactured by an artisan producer had the statistically highest (poly)phenol content and antioxidant capacity by ABTS assay. With the exception of an apple sauce manufactured by an artisan producer, estimation of total (poly)phenols and antioxidant capacity were significantly higher in apple sauce products when compared to jellies or chutneys. ^o^Brix, an indication of sugar content was highest in apple butter—artisan with apple sauce products lower than jellies, preserves and chutneys. (Poly)phenols are known to be more stable in acidic conditions^34^ and two apple sauce products (one branded and one supermarket own-brand) had a lower pH than other products.

**Table 1:** Initial screening—Comparison of products available in NI.

Product	Package description of apple ingredient	^o^Brix	pH	Dry matter %	TP assay (mg GAE kg^−1^ FW)	DPPH (mmol TROLOX eq kg^−1^FW)	ABTS (mmol TROLOX eq kg^−1^FW)
Apple jelly 1	Apple juice	58.5 ± 0.0f.	2.8 ± 0.1^c^	62.2 ± 0.7f.	1203 ± 21^c^	7.48 ± 0.49^ef^	1.48 ± 0.06^a^
Apple jelly 2	Apples	61.3 ± 0.6^ h^	3.0 ± 0.1^de^	64.6 ± 1.1^ g^	872 ± 74^b^	5.28 ± 0.29^c^	0.97 ± 0.10^a^
Chilli apple jelly	Apple juice	60.3 ± 0.3^gh^	3.1 ± 0.1^ef^	63.9 ± 0.4^ fg^	1448 ± 34^d^	7.80 ± 0.28f.	1.65 ± 0.04^a^
Clove apple jelly	Apples	60.3 ± 0.3^gh^	2.9 ± 0.1^cde^	64.4 ± 0.7^ fg^	1185 ± 13^c^	7.34 ± 0.40^ef^	1.47 ± 0.06^a^
Spiced apple & fig preserve	Apples	59.8 ± 1.9^ g^	3.5 ± 0.1^gh^	64.1 ± 2.0^ fg^	645 ± 4^a^	2.84 ± 0.05^a^	1.48 ± 0.14^a^
Apple & blueberry preserve	Bramley	62.8 ± 1.4^i^	2.9 ± 0.1^ cd^	67.1 ± 2.5^ h^	1089 ± 49^c^	5.36 ± 0.25^c^	4.88 ± 0.20^bc^
Spiced apple chutney	Apples	36.7 ± 0.3^b^	3.6 ± 0.0^hi^	39.0 ± 0.3^b^	1122 ± 54^c^	4.04 ± 0.14^b^	4.46 ± 0.41^b^
Plum and apple chutney	Armagh Bramley	49.5 ± 0.5^e^	3.5 ± 0.1^ g^	51.4 ± 1.3^e^	1729 ± 60^e^	6.01 ± 0.10^ cd^	8.26 ± 0.36^d^
Apple butter	Armagh Bramley	74.0 ± 0.5^j^	3.7 ± 0.0^i^	78.4 ± 0.4^i^	3613 ± 48^ h^	18.79 ± 0.63^ h^	24.20 ± 2.73^ h^
Apple sauce 1	Armagh Bramley	38.8 ± 0.8^c^	2.9 ± 0.2^ cd^	41.8 ± 0.4^c^	1084 ± 203^c^	6.67 ± 1.63^de^	6.34 ± 2.27^c^
Apple sauce 2	Bramley	32.5 ± 0.0^a^	2.5 ± 0.0^a^	32.2 ± 3.0^a^	2061 ± 41f.	17.66 ± 0.62^ g^	16.31 ± 0.32^e^
Apple sauce 3	British Bramley	31.3 ± 0.3^a^	3.2 ± 0.0f.	33.6 ± 0.4^a^	1949 ± 41f.	18.70 ± 0.41^ h^	18.34 ± 0.55f.
Apple sauce 4	Kent Bramley	41.8 ± 0.3^d^	2.6 ± 0.0^b^	44.5 ± 0.2^d^	2193 ± 93^ g^	21.26 ± 0.52^i^	20.43 ± 0.27^ g^

Data expressed as mean ± SD (n = 3). Within columns different letters denote significant differences between groups (*p* < 0.05; one-way ANOVA, Fisher’s least significance test).

### Targeted investigation: raw material

Total (poly)phenol content, ascorbic acid and antioxidant content for apple raw material used to prepare seven products are detailed in Table 2. Total (poly)phenol ranged from 1610 to 2650 mg kg^–1^ FW in raw material. Proanthocyanidins were the highest contributor (~ 64%) to (poly)phenolic content, flavanols accounted for ~ 16% and phenolic acids contributed ~ 17%. Dihydrochalcones and flavonols each accounted for between ~ 1 and 3%. Cyanidin 3-galactoside was not found in raw materials. Ascorbic acid ranged from 92.4 to 745.4 mg kg^–1^ FW.

**Table 2:** (Poly)phenol, ascorbic acid, dry matter and antioxidant content in raw materials used to prepare seven Armagh Bramley apple products.

Raw Material (mg kg^−1^ FW)	Diced apple raw material	Apple puree raw material	Canned sliced apple raw material	Apple sauce -industrial raw material	Apple butter - artisan raw material	NPD (sauce/apple butter) raw material
Procyanidin B1	73 ± 5.2	86.4 ± 11.5	75.9 ± 11.7	83.0 ± 11.7	68.7 ± 21.8	95.9 ± 17.1
Procyanidin B2	174 ± 13.2	149 ± 30.3	176 ± 34.7	133.2 ± 42.9	118 ± 27.4	186 ± 11.7
Catechin	28.8 ± 4.3	25.1 ± 6.2	30.7 ± 9.4	22.1 ± 10.1	23.3 ± 5.3	40.2 ± 2.4
Epicatechin	124 ± 18.4	97.2 ± 28.5	119 ± 29.0	79.4 ± 29.2	76.9 ± 16.2	83.8 ± 8.5
**∑ Flavanols**	**401 ± 37.9**	**358 ± 60.2**	**402 ± 80.8**	**317.8 ± 79.4**	**286 ± 57.8**	**406 ± 38.3**
Total Proanthocyanidins	1447 ± 178	1458 ± 104	1571 ± 236	1373 ± 596.6	939 ± 106	1747 ± 223
DPn	4.1 ± 0.6	4.8 ± 0.2	3.8 ± 0.9	4.5 ± 0.8	4.1 ± 0.4	3.9 ± 0.2
Chlorogenic acid	413 ± 44.9	326 ± 82.8	360.0 ± 52.7	294.1 ± 105	287.4 ± 63.3	393 ± 14.3
p-Coumaroylquinic acid	46.4 ± 1.2^b^	31.6 ± 5.7^a^	43.2 ± 6.1^b^	27.0 ± 6.4^a^	27.9 ± 8.6^a^	30.1 ± 3.1^a^
**∑ Phenolic acids**	**459 ± 44.5**	**358 ± 88.5**	**403 ± 58.8**	**321.1 ± 111.3**	**315 ± 71.7**	**423 ± 17.5**
Phloretin xyloglucoside	21.3 ± 1.7	21.2 ± 1.4	17.7 ± 2.8	19.8 ± 9.3	18.9 ± 1.4	19.0 ± 1.0
Phlorizin	33.5 ± 4.6	27.0 ± 7.9	28.3 ± 2.8	30 ± 30.3	44.0 ± 30	21.7 ± 2.7
**∑ Dihydrochalcones**	**54.8 ± 5.8**	**48.2 ± 8.8**	**46.1 ± 5.3**	**49.8 ± 39.5**	**62.8 ± 4.3**	**40.7 ± 2.7**
Quercetin 3-rutinoside	0.8 ± 0.8	0.6 ± 0.5	1.0 ± 0.7	0.2 ± 0.3	0.4 ± 0.4	2.2 ± 2.3
Quercetin 3-galactoside	6.4 ± 4.1	8.1 ± 2.6	6.7 ± 3.3	5.4 ± 2.0	4.2 ± 2.5	13.7 ± 9.3
Quercetin 3-glucoside	1.0 ± 0.3	2.2 ± 0.9	2.2 ± 0.5	1.7 ± 1.2	1.0 ± 1.0	4.8 ± 3.0
Quercetin 3-rhamnoside	4.1 ± 1.3^a^	5.8 ± 1^ab^	5.1 ± 0.4^a^	4.8 ± 0.8^a^	3.0 ± 1.3^a^	8.8 ± 3.7^b^
**∑ Flavonols**	**12.4 ± 4.7**	**16.7 ± 4.7**	**15.0 ± 4.5**	**12.1 ± 4.3**	**8.6 ± 4.9**	**29.5 ± 18.1**
**∑ (poly)phenol**	**2374 ± 178**	**2239 ± 237**	**2437 ± 350**	**2074 ± 823.5**	**1613 ± 213**	**2647 ± 244**
Ascorbic acid	92.4 ± 34.9	137.2 ± 29.4	97.5 ± 49.0	95.7 ± 18.7	745.4 ± 640.9	141.6 ± 38.1
TP assay (mg GAE kg^−1^ FW)	1372 ± 92	1880 ± 655	1339 ± 143	1295 ± 328	1411 ± 269	1266 ± 51
DPPH (mmol TROLOX eq kg^−1^FW)	9.17 ± 0.29	8.76 ± 0.48	9.02 ± 0.92	8.43 ± 1.25	9.67 ± 2.04	8.84 ± 1.05
ABTS (mmol TROLOX eq kg^−1^FW)	12.4 ± 0.3	10.9 ± 1.2	11.4 ± 1.0	10.4 ± 2.9	11.6 ± 2.7	10.8 ± 11.6
Dry matter (%)	12.9 ± 0.8^b^	12.6 ± 1.0^b^	12.7 ± 1.1^b^	13.1 ± 0.9^bc^	9.9 ± 0.8^a^	14.5 ± 0.2^c^

Data expressed ± standard deviation (n = 3). Within rows different letters denote significant differences between groups (*p* < 0.05; one-way ANOVA, Fisher’s least significance test). Sum of (poly)phenols by structural group are in bold. *DPn* degree of polymerisation of Proanthocyanidins. Raw materials were apple except for apple butter—artisan produced from puree. Both NPD products were prepared using the same raw material.

Estimation of total phenols (TP) by assay ranged from 1266 to 1880 mg GAE eq kg^–1^ FW with antioxidant capacity ranging from 8.43 to 9.67 mmol TROLOX eq kg^–1^ FW and 10.4 to 12.4 mmol TROLOX eq kg^–1^ FW by DPPH and ABTS assays respectively. There were no significant differences observed for (poly)phenol structural groups or antioxidant content (TP, ABTS, DPPH) between raw materials. Significant differences (*p* < 0.001) were observed for dry matter, which ranged from 12.7 to 14.5% in whole apples, while apple butter – artisan puree raw material was significantly lower at 9.9%.

### Targeted investigation: products

Total (poly)phenol content, ascorbic acid, antioxidant content as well as percentage change on comparison to raw materials and nutritional data for seven products are detailed in Table 3A, B. Total (poly)phenol content ranged from 726 to 1919 mg kg^–1^ FW. When content of products was compared to raw material, total (poly)phenolic content was reduced in all products on processing in the order apple butter—artisan (55.0%) > dice (51.3%) > apple butter NPD (45.9%) > canned sliced apple (45.1%) > sauce NPD (44.8%) > puree (14.6%) > industrial sauce (7.5%). When products were compared statistical differences for total (poly)phenols were observed (*p* = 0.009) in the mean product order of industrial sauce > puree > sauce NPD > apple butter NPD > canned sliced apple > dice > apple butter—artisan. Proanthocyanidins remained the highest contributor to total (poly)phenols after processing contributing between 43.7 and 61.9%, with all products reduced in these compounds when compared to raw material. The largest proanthocyanidin processing loss was observed in apple butter—artisan (66.2%), whereas in the industrial intermediate products puree and sauce, minor losses of 18.9% and 15.2% respectively were found. Significant differences between proanthocyanidin content of products (*p* = 0.011) were observed in the mean order puree > industrial sauce > canned sliced apple > sauce NPD > apple butter NPD > dice > apple butter – artisan.

**Table 3:** Comparison of Armagh Bramley products in terms of (poly)phenol, ascorbic acid, dry matter, antioxidant and nutritional content. Direction of percentage change (%) from raw material indicated by arrows. Table A denotes content, and B denotes % change from raw materials and nutritional content of products.

A Product (mg kg^−1^ FW)	Diced apple product	Apple puree product	Canned sliced apple product	Apple sauce—industrial product	Apple butter—artisan product	Apple sauce—NPD product	Apple butter—NPD product
Procyanidin B1	38.2 ± 19.9^a^	62.9 ± 4.6^a^	48.6 ± 1.2^a^	62.6 ± 26.9^a^	154.0 ± 97.4^b^	78.8 ± 16.3^a^	33.2 ± 2.9^a^
Procyanidin B2	61.2 ± 48.1^b^	149 ± 8.3^e^	101 ± 16.8^bcd^	130 ± 42.0^de^	8.4 ± 14.6^a^	79.2 ± 15.6^bc^	109 ± 4.5^cde^
Catechin	13.7 ± 14.8^ab^	19.2 ± 2.4^abc^	16.7 ± 2.9^ab^	25.4 ± 8.2^bcd^	7.1 ± 8.5^a^	38.1 ± 4.7^d^	30.9 ± 2.0^ cd^
Epicatechin	50.6 ± 41.4^b^	91.9 ± 10.3^d^	61.4 ± 8.0^bcd^	84.3 ± 21.6^ cd^	< LOD^a^	56.5 ± 8.0^bc^	51.5 ± 9.1^bc^
**∑ Flavanols**	**164 ± 124.1**	**323 ± 15.1**	**228 ± 22.9**	**302 ± 81.7**	**170 ± 119.5**	**253 ± 44.5**	**224 ± 17.4**
Total Proanthocyanidins	648 ± 102.3^ab^	1183 ± 163.7^d^	769 ± 54.5^bcd^	1164 ± 415.0^ cd^	317 ± 304.1^a^	758 ± 353.2^bcd^	739 ± 44.6^abc^
DPn	4.0 ± 0.7^b^	4.5 ± 0.6^b^	3.6 ± 0.3^b^	3.8 ± 0.4^b^	< LOD^a^	2.0 ± 1.6^b^	3.7 ± 0.5^c^
Chlorogenic acid	280 ± 162.4	248 ± 45.4	283 ± 9.9	329 ± 104.7	170 ± 28.3	376 ± 33.1	356 ± 16.2
p-Coumaroylquinic acid	33.5 ± 8.7^bc^	21.4 ± 4.5^a^	29.4 ± 4.5^ab^	30.1 ± 7.8^abc^	37.6 ± 9.5^bc^	40.6 ± 4.0^c^	30.1 ± 1.7^abc^
**∑ Phenolic acids**	**314 ± 171.0**	**270 ± 49.9**	**313 ± 11.6**	**359 ± 112.3**	**208 ± 34.1**	**416 ± 36.4**	**386 ± 17.8**
Phloretin xyloglucoside	10.6 ± 2.4^a^	31.9 ± 0.8^c^	11.5 ± 1.1^a^	26 ± 6.4^b^	9.0 ± 4.2^a^	12.4 ± 1.3^a^	21.5 ± 1.1^b^
Phlorizin	20.7 ± 1.8^a^	68.5 ± 5.8^c^	16.6 ± 2.8^a^	53.5 ± 19.1^b^	22.6 ± 1.6^a^	21.2 ± 1.9^a^	23.9 ± 0.4^a^
**∑ Dihydrochalcones**	**31.3 ± 2.9**^**a**^	**100 ± 5.1**^**c**^	**28.1 ± 3.9**^**a**^	**79.5 ± 25.1**^**b**^	**31.6 ± 5.5**^**a**^	**33.6 ± 1.8**^**a**^	**45.5 ± 1.5**^**a**^
Quercetin 3-rutinoside	< LOD^a^	2.3 ± 0.2^b^	< LOD^a^	0.6 ± 0.7^a^	0.5 ± 0.9^a^	< LOD^a^	3.7 ± 1.2^c^
Quercetin 3-galactoside	< LOD^a^	15.3 ± 0.8^c^	< LOD^a^	6.6 ± 3.9^b^	0.2 ± 0.4^a^	< LOD^a^	18.5 ± 4.3^c^
Quercetin 3-glucoside	< LOD^a^	5.6 ± 0.9^c^	< LOD^a^	2.4 ± 1.6^b^	< LOD^a^	< LOD^a^	6.8 ± 2.2^c^
Quercetin 3-rhamnoside	< LOD^a^	10.9 ± 1.3^c^	< LOD^a^	5 ± 2.3^b^	< LOD^a^	< LOD^a^	9.5 ± 1.0^c^
**∑ Flavonols**	** < LOD**^**a**^	**34.1 ± 2.8**^**c**^	** < LOD**^**a**^	**14.6 ± 8.4**^**b**^	**0.8 ± 0.8**^**a**^	** < LOD**^**a**^	**38.4 ± 8.5**^**c**^
**∑ (poly)phenol**	**1157 ± 398**^**ab**^	**1911 ± 211**^**c**^	**1338 ± 55**^**bc**^	**1919 ± 626**^**c**^	**726 ± 323**^**a**^	**1460 ± 291**^**bc**^	**1433 ± 85**^**bc**^
Ascorbic acid	2.7 ± 2.4^a^	44.4 ± 8^a^	24.5 ± 15.6^a^	18.1 ± 4.5^a^	335 ± 150^b^	43.1 ± 11^a^	47.6 ± 3.9^a^
TP assay (mg GAE kg^−1^ FW)	806 ± 218^a^	1246 ± 156^b^	798 ± 37^a^	1279 ± 359^b^	3353 ± 280^d^	1104 ± 114^ab^	1904 ± 62^c^
DPPH (mmol TROLOX eq kg^−1^FW)	5.59 ± 1.83^a^	7.40 ± 0.14^ab^	5.27 ± 0.10^a^	7.77 ± 1.63^ab^	17.8 ± 2.6^d^	8.63 ± 1.08^bc^	10.8 ± 1.1^c^
ABTS (mmol TROLOX eq kg^−1^FW)	6.40 ± 2.96^a^	9.47 ± 1.63^bc^	6.77 ± 0.38^ab^	9.73 ± 2.54^c^	21.9 ± 0.5^e^	10.6 ± 0.6^ cd^	13.4 ± 1.1^d^
Dry matter (%)	11.8 ± 1.1^b^	7.8 ± 1^a^	8.6 ± 0.5^a^	10.7 ± 0.4^b^	74 ± 0.5^e^	27.2 ± 1.9^c^	32.7 ± 0.8^d^

B. Product (% change from raw material)	Diced apple product	Apple puree product	Canned sliced apple product	Apple sauce—industrial product	Apple butter—artisan product	Apple sauce – NPD product	Apple butter – NPD product
Procyanidin B1	↓47.7	↓27.2	↓35.9	↓24.6	↑124	↓17.8	↓65.4
Procyanidin B2	↓64.9	↓0.0	↓42.4	↓2.7	↓92.9	↓57.5	↓41.6
Catechin	↓52.6	↓23.5	↓45.7	↑14.8	↓69.5	↓5.3	↓23.2
Epicatechin	↓59.3	↓5.4	↓48.7	↑6.2	↓100	↓32.6	↓38.5
**∑ Flavanols**	↓**59.1**	↓**9.7**	↓**43.3**	↓**5.0**	↓**40.8**	↓**37.8**	↓**44.8**
Total Proanthocyanidins	↓55.2	↓18.9	↓51.1	↓15.2	↓66.2	↓56.6	↓57.7
DPn	↓1.1	↓5.6	↓4.7	↓14.9	↓100	↓48.2	↓6.0
Chlorogenic acid	↓32.2	↓23.9	↓21.4	↑11.9	↓40.7	↓4.5	↓9.4
p-Coumaroylquinic acid	↓27.7	↓32.3	↓31.9	↑11.8	↓34.9	↓35.0	↑0.1
**∑ Phenolic acids**	↓**31.7**	↓**24.7**	↓**22.5**	↑**11.9**	↓**34.0**	↓**1.7**	↓**8.7**
Phloretin xyloglucoside	↓50.4	↑50.5	↓35.2	↑31.4	↓52.4	↓34.8	↑13.4
Phlorizin	↓38.1	↑154	↓41.6	↑78.0	↓48.5	↓2.1	↑10.3
**∑ Dihydrochalcones**	↓**42.9**	↑**108**	↓**39.0**	↑**59.6**	↓**49.7**	↓**17.4**	↑**11.8**
Quercetin 3-rutinoside	↓100	↑299	↓100	↑182	↑26.5	↓100	↑38.2
Quercetin 3-galactoside	↓100	↑89.9	↓100	↑21.3	↓94.2	↓100	↑34.4
Quercetin 3-glucoside	↓100	↑150	↓100	↑42.5	↓100	↓100	↑41.3
Quercetin 3-rhamnoside	↓100	↑88.2	↓100	↑4.5	↓100	↓100	↑7.8
**∑ Flavonols**	↓**100**	↑**104**	↓**100**	↑**20.5**	↓**91.0**	↓**100**	↑**30.0**
**∑ (poly)phenol**	↓**51.3**	↓**14.6**	↓**45.1**	↓**7.5**	↓**55.0**	↓**44.8**	↓**45.9**
Ascorbic acid	↓97.1	↓67.6	↓74.9	↓81.1	↓55.1	↓69.6	↓66.4
TP assay	↓41.3	↓33.7	↓40.4	↓1.2	↑138	↓12.8	↑50.4
DPPH	↓39.1	↓15.5	↓41.5	↓7.9	↑84.5	↓2.4	↑22.6
ABTS	↓48.6	↓13.0	↓40.4	↓6.7	↑88.0	↓1.4	↑25.0
Dry matter (%)	↓8.7	↓38.6	↓32.3	↓18.1	↑649	↑88.3	↑126
**Nutrition/100 g**							
Energy (kj)	168.5	162.2	160.5	159.6	1350	466.4	493.4
Fat (g)	0.0	0.0	0.0	0.0	0.5	4.1	0.3
Saturates (g)	0.0	0.0	0.0	0.0	< 0.1	3.2	0.1
Carbohydrate (g)	9.6	7.2	9.2	9.1	75.1	18.8	28.4
Sugars (g)	9.2	6.9	8.9	8.8	73.7	18.8	28.3
Fibre (g)	2.4	1.8	1.6	1.6	2.3	1.7	2.3
Protein (g)	0.3	0.3	0.2	0.2	0.8	0.2	0.2
Salt (g)	0.01	0.0	0.0	0.0	0.3	0.0	0.5

Data expressed mean ± standard deviation (n = 3). Within rows different letters denote significant differences between groups (*p* < 0.05; one-way ANOVA, Fisher’s least significance test). Sum of (poly)phenols by structural group are in bold. *DPn* degree of polymerisation of Proanthocyanidins.

↑Increment vs. raw material; ↓diminution vs. raw material. Nutritional data either received from producer or derived using McCance and Widdowson database^35^.

Phenolic acids contributed between 23.4 and 28.7% of total (poly)phenolic content for diced apple, canned sliced apple, apple butter—artisan and both NPDs, with lower percentages for apple puree and industrial apple sauce at 14.1 and 18.7% respectively. Phenolic acids increased on processing for industrial sauce (11.9%), with a reduction observed for all other products in the mean order apple butter—artisan (34%) > dice (31.7%) > puree (24.7%) > canned sliced apple (22.5%) > apple butter NPD (8.7%) > sauce NPD (1.7%). No significant differences were observed between products for the sum of phenolic acids, although a difference (*p* = 0.049) was observed for p-coumaroylquinic acid.

Total flavanols contributed 14.1–23.3% to total (poly)phenols in products and this structural group was reduced on processing for all products. Reductions were in the order of diced (59.1%) > apple butter NPD (44.8%) > canned sliced apple (43.3%) > apple butter—artisan (40.8%) > sauce NPD (37.8%) > puree (9.7%) > industrial sauce (5%). Notably some individual compounds increased on processing. For example, in industrial sauce, catechin and epicatechin increased (14.8 and 6.2% respectively), while procyanidin B1 increased in apple butter—artisan (124.2%). No statistical differences were observed between the products for the flavanol group; however, differences were observed for individual compounds (procyanidin B1, *p* = 0.033; catechin, *p* = 0.003; procyanidin B2, *p* < 0.001; epicatechin, *p* < 0.001).

Dihydrochalcones contributed between 2.1% and 5.3% to products, with increases on processing when compared to raw material observed for apple butter NPD (11.8%), industrial sauce (59.6%) puree (108.3%), while reductions were observed for other products in the order and apple butter—artisan (49.7%) > dice (42.9%) > canned sliced apple (39.0%) > sauce NPD (17.4%). Statistical differences (*p* < 0.001) were observed for this structural group and both contributing dihydrochalcones measured in the mean order of puree > industrial sauce > butter NPD > sauce NPD > apple butter—artisan > dice > canned sliced apple.

Flavonols were only greater than the limit of detection in products which had been cooked with the peel included, with contributions ranging from 0.1% to 2.7%. With the exception of apple butter—artisan, proportional contributions of individual compounds were observed to be in the same order as in whole apple, quercetin 3-galactoside > quercetin 3-rhamnoside > quercetin 3-glucoside > quercetin 3-rutinoside. In comparison with raw material, increases were observed in industrial sauce (20.5%), apple butter NPD (30.0%) and puree (104.4%). A reduction on processing was observed for apple butter—artisan (91.1%) although quercetin 3-rutinoside increased by 26.5% in this product. Statistical differences were observed for this structural group as well as each of the four flavonols measured (*p* < 0.001) in the mean order of apple butter NPD > puree > industrial sauce > apple butter—artisan. Cyanidin 3-galactoside was only observed in the apple puree samples contributing 0.003% to the total. This compound was below the LOD in the raw material.

Ascorbic acid ranged from 2.7 to 334.7 mg kg^–1^ FW. Ascorbic acid was reduced in all products on processing, the largest reductions were observed in diced apple (97.1%), industrial sauce (81.1%) and canned sliced apple (74.9%). Sauce NPD, apple butter NPD and puree were reduced by similar amounts of 69.6%, 66.4% and 67.6% respectively. Statistical differences (*p* < 0.001) observed between products were in the mean order apple butter—artisan > apple butter NPD > puree > sauce NPD > canned sliced apple > industrial sauce > dice.

Estimation of total phenols by assay ranged from 798 to 3353 mg GAE eq kg^–1^ FW with antioxidant capacity ranging from 5.27 to 17.8 mmol TROLOX eq kg^−1^ FW and 6.40 to 21.9 mmol TROLOX eq kg^–1^ FW by DPPH and ABTS assays respectively. Apple butter—artisan demonstrated the highest value for all three assays. Statistical differences were observed between products for all assays (*p* < 0.001). Products analysed by both ABTS and DPPH followed the mean order, apple butter—artisan > apple butter NPD > sauce NPD > industrial sauce > puree > canned sliced apple > dice. While estimation of total (poly)phenols by assay followed the mean order, apple butter—artisan > apple butter NPD > industrial sauce > sauce NPD > puree > diced apple > canned sliced apple.

### Targeted investigation: pomace

Total (poly)phenol content, ascorbic acid and antioxidant content remaining in pomace following processing are detailed in Table 4 along with the percentage of raw material it represents. Total (poly)phenolic content ranged from 1974 to 5588 mg kg^–1^ FW with statistical differences observed (*p* < 0.001) and in the mean order of pomace from industrial sauce > sauce NPD > puree > canned sliced apple > apple butter NPD > dice.

**Table 4:** Comparison of apple pomace generated from the processing of Armagh Bramley apple products in terms of (poly)phenol, ascorbic acid, dry matter and antioxidant content.

Pomace (mg kg^−1^ FW)	Diced apple pomace	Apple puree pomace	Canned sliced apple pomace	Apple sauce – industrial pomace	Apple sauce -NPD pomace	Apple butter – NPD pomace
Procyanidin B1	27.5 ± 5.7^a^	56.6 ± 10.3^c^	37.1 ± 5.4^ab^	93.2 ± 21.0^d^	80.4 ± 14.4^d^	58.9 ± 1.5^c^
Procyanidin B2	90.8 ± 16.2^a^	193 ± 40.5^b^	161 ± 10.7^b^	266 ± 82.3^c^	331 ± 13.8^c^	164 ± 5.3^b^
Catechin	11.6 ± 1.8^a^	17.0 ± 1.6^a^	12.9 ± 3.6^a^	33.4 ± 14.6^b^	53.2 ± 4.2^c^	39.9 ± 0.5^b^
Epicatechin	66.7 ± 13.0^a^	122 ± 25.7^bc^	97.3 ± 1.4^ab^	159 ± 38.3^d^	156 ± 10.0^ cd^	80.6 ± 1.4^a^
**∑ Flavanols**	**197 ± 36.0**^**a**^	**388 ± 69.8**^**b**^	**308 ± 1.3**^**ab**^	**552 ± 143.9**^**c**^	**620 ± 29.8**^**c**^	**343 ± 6.7**^**b**^
Total Proanthocyanidins	1339 ± 218^a^	2819 ± 615^c^	2426 ± 293^bc^	4156 ± 710^d^	3739 ± 243^d^	1804 ± 18^ab^
DPn	4.7 ± 0.7^a^	5.4 ± 0.1^a^	5.1 ± 0.4^a^	6.4 ± 1.1^b^	4.9 ± 0.0^a^	5.1 ± 0.3^a^
Chlorogenic acid	199 ± 47.4^a^	244 ± 88.9^ab^	144 ± 26.5^a^	365 ± 113.8^c^	326 ± 22.4^bc^	358 ± 13.2^c^
p-Coumaroylquinic acid	20.1 ± 4.7^ab^	24.7 ± 8.3^ab^	19.1 ± 5.7^ab^	34.6 ± 5.8^c^	17.6 ± 1.8^a^	27.9 ± 0.5^bc^
**∑ Phenolic acids**	**219 ± 52.1**^**a**^	**269 ± 96.9**^**ab**^	**163 ± 32.2**^**a**^	**400 ± 119.6**^**c**^	**344 ± 21.9**^**bc**^	**386 ± 13.6**^**bc**^
Phloretin xyloglucoside	39.2 ± 6.6^b^	52.9 ± 2.4^c^	26.6 ± 2.0^a^	79.0 ± 11.7^d^	70.0 ± 5.5^d^	19.7 ± 1.7^a^
Phlorizin	134 ± 16.9^b^	131 ± 37.3^b^	61.7 ± 3.3^ab^	315 ± 102.8^c^	129 ± 15.7^b^	25.6 ± 0.7^a^
**∑ Dihydrochalcones**	**173 ± 23.2**^**b**^	**184 ± 39.5**^**b**^	**88.4 ± 4.1**^**a**^	**394 ± 91.1**^**c**^	**199 ± 20.8**^**b**^	**45.2 ± 2.2**^**a**^
Quercetin 3-rutinoside	3.5 ± 1.3^a^	7.0 ± 3.3^ab^	6.5 ± 3.2^a^	5.0 ± 2.3^a^	11.8 ± 4.3^b^	4.1 ± 1.2^a^
Quercetin 3-galactoside	20.2 ± 5.5^a^	46.6 ± 14.4^a^	37.9 ± 18.7^a^	36.7 ± 14.2^a^	85.0 ± 23.9^b^	23.4 ± 4.5^a^
Quercetin 3-glucoside	7.2 ± 2.0^a^	17.6 ± 3.4^a^	13.6 ± 6.0^a^	14.4 ± 5.2^a^	36.3 ± 11.2^b^	8.7 ± 2.2^a^
Quercetin 3-rhamnoside	14.9 ± 3.8^a^	36.9 ± 7.0^b^	30.9 ± 13.3^b^	29.7 ± 6.9^b^	51.2 ± 7.4^c^	12.1 ± 1.3^a^
**∑ Flavonols**	**45.7 ± 12.4**^**a**^	**108 ± 27.7**^**b**^	**88.8 ± 41.0**^**ab**^	**85.8 ± 28.0**^**ab**^	**184 ± 46.0**^**c**^	**48.2 ± 9.1**^**a**^
Cyanidin 3-galactoside	< LOD	0.1 ± 0.3	< LOD	< LOD	< LOD	< LOD
**∑ (poly)phenol**	**1974 ± 325**^**a**^	**3769 ± 839**^**c**^	**3074 ± 290**^**bc**^	**5588 ± 898**^**d**^	**5087 ± 274**^**d**^	**2627 ± 12**^**ab**^
Ascorbic acid	1.1 ± 1.9^a^	43.9 ± 24.5^bc^	14.7 ± 25.5^ab^	35.7 ± 24.2^abc^	125.2 ± 28.8^d^	61.3 ± 11.1^c^
TP assay (mg GAE kg^−1^ FW)	1025 ± 206^a^	1972 ± 432^b^	1380 ± 216^a^	2308 ± 490^b^	1969 ± 52^b^	1275 ± 158^a^
DPPH (mmol TROLOX eq kg^−1^FW)	6.43 ± 1.09^a^	11.8 ± 2.3^c^	9.26 ± 1.02^b^	16.7 ± 1.4^d^	14.1 ± 0.6^c^	8.55 ± 0.8^ab^
ABTS (mmol TROLOX eq kg^−1^FW)	8.18 ± 1.47^a^	15.1 ± 4.5^c^	11.3 ± 1.3^ab^	19.0 ± 4.3^c^	19.5 ± 0.6^c^	11.1 ± 1.1^ab^
Dry matter (%)	10.5 ± 0.5^a^	15.1 ± 1.0^abc^	11.2 ± 0.4^ab^	24.0 ± 6.3^d^	17.7 ± 0.6^c^	15.8 ± 0.3^bc^
Pomace yield (%)	40–45	2–4	30–35	2–4	25–30	20–25

Results expressed ± standard deviation (n = 3). Within rows different letters denote significant differences between groups (*p* < 0.05; one-way ANOVA, Fisher’s least significance test). Sum of (poly)phenols by structural group are in bold. *DPn* degree of polymerisation of Proanthocyanidins. Apple butter—artisan processing did not result in any pomace produced as the product was prepared from apple puree.

Proanthocyanidins were the highest contributor (67.8–78.9%) to total (poly)phenol content, flavanols contributed between 9.9 and 13.1%, with phenolic acids contributing a similar range of 5.3–14.7%. Dihydrochalcones and flavonols were found in similar proportions of 1.7–8.8% and 1.5–3.6%. Cyanidin 3-galactoside was only found in the puree product pomace in small concentrations and in only one collection. Statistical differences were observed between pomace for all structural groups (flavanols, *p* < 0.001; phenolic acids, *p* = 0.006; dihydrochalcones, *p* < 0.001; flavonols, *p* = 0.001) as well as all individual compounds measured.

Ascorbic acid ranged from 1.1 to 125 mg kg^–1^ FW with statistical differences observed (*p* < 0.001) as shown in Table 4 in the mean order of pomace from sauce NPD > butter NPD > puree > industrial sauce > canned sliced apple > diced apple.

Estimation of total phenols in pomace by assay ranged from 1025 to 2308 mg GAE eq kg^–1^ FW with antioxidant capacity ranging from 6.43 to 16.7 mmol TROLOX eq kg^–1^ FW and 8.18 to 19.5 mmol TROLOX eq kg^–1^ FW by DPPH and ABTS assays respectively. Statistical differences were observed for all assays between pomace (DPPH, *p* < 0.001; ABTS, *p* = 0.001; TP, *p* = 0.001) and pomace from diced apple contained the lowest capacity in all cases.

## Discussion

This targeted investigation assessed and compared the (poly)phenolic and antioxidant content of Armagh Bramley products and pomace. Results indicated that there were no substantial differences between raw materials. The products containing the highest (poly)phenol content were industrially produced apple puree and sauce. Pomace contained significant (poly)phenol content and antioxidants.

### Targeted investigation: raw material

The (poly)phenol content of raw materials in the study analysis is in good agreement with a previous study where an average total (poly)phenol value of 16.4 g kg^−1^ DW (2476 mg kg^−1^ FW) was reported for a group containing both Bramley and Armagh Bramley apples^14^. Ascorbic acid content found in the whole apple raw materials is also in good agreement to previous measurements^31^. It was noted that this compound was particularity high (1479 mg kg^–1^ FW) in one collection of apple butter—artisan raw material and may have been added as an antioxidant by the producer. As there were no substantive differences between raw materials, it can be ascertained that any differences between processed products are a result of processing.

### Targeted investigation: products

To the authors knowledge this is the first time a variety of products made using different processing techniques from minimal to intensive, have been considered in one study, with data essential to inform industry on ways to maximise nutraceutical content of final products. The (poly)phenolic content from all products in the current study was higher than studies including both apple puree and apple sauce produced from other apple cultivars^15,36^ while being in good agreement with a study describing the effects of prebiotic additions to Bramley’s seedling apple purees^37^, suggesting that products produced using Armagh Bramley’s have similar bioactive content.

Retention of (poly)phenolics in the final products was affected by the type of processing used, with differences also observed in the profile of products which included peel and core. Flavonols were below the limit of detection in products which were not processed with the inclusion of peel, whereas increases in these compounds on processing were observed in puree (104%), industrial apple sauce (20.5%) and apple butter NPD (30%). The highest increase in dihydrochalcones, also known to be high in skin and particularly core^38^ were observed in puree (108%) which included skin and core during production. This is consistent with a study of industrially produced apple puree, although increases reported in other (poly)phenols were not observed in the current study^39^. More moderate increases were observed in products which had been cored but not peeled (industrial sauce and apple butter NPD), with a higher increase observed in the industrial product. Those products that did not include either core or peel contained a lower flavonol content post-processing (dice, apple sauce NPD and apple butter—artisan). These results indicate a transfer of flavonols and dihydrochalcones from peel/core to product during production.

Differences in thermal processing may also help explain variations in products with the highest mean total (poly)phenol content found in industrial products subjected to ‘screw cooking’ (puree and industrial sauce) containing similar concentrations of many individual compounds. In whole apple tissue (poly)phenols are contained within separate vacuoles and when subjected to mechanical disruption (poly)phenols can react with cell wall polysaccharides and be oxidised by (poly)phenol oxidase (PPO)^40^, which is deactivated on exposure to high temperatures^41^. Screw cooking (extrusion) is a thermomechanical process commonly used in industrially produced products, where apple tissue is subjected to mechanical destruction whilst being simultaneously subjected to heat, leading to shorter processing times^42^. The rapid nature of extrusion processing may explain the higher retention of some (poly)phenolic compounds in puree and industrial sauce, as demonstrated by a study investigating apple destructuring alongside with thermal treatments^43^. It has also been demonstrated that sodium chloride can suppress PPO activity^44^ and it should be noted that apples used for the production of both canned sliced apple and industrial apple sauce were stored in a brine solution prior to heat processing, which may have affected PPO activity. The lowest total mean (poly)phenol content in the industrial products was reported in diced apple which had not been subjected to thermal processing, with PPO consequently not denatured in this product. In contrast the extensive thermal processing, which apple butter—artisan (lowest mean total (poly)phenols) was subjected to, may have ultimately destructured many (poly)phenolics including proanthocyanidins. It was noted that following acid-catalysed depolymerisation, phloroglucinol adducts were not present, with only existing monomers and terminal subunits measured in the proanthocyanidin analysis. This suggests that only flavanol monomers and dimers remained post process for this product. This may explain the significantly higher levels of procyanidin B1 when compared to raw material, a possible breakdown product from larger proanthocyanidins and other dimers not determined by analyses carried out in the current study may be also present in this product. While most studies on apples have been carried out on minimally processed products, studies on fruit jams have suggested that the rate of degradation varies with intensity of process and losses of up to 89% of proanthocyanidins have been reported^45^.

The addition of non-apple material, such as sugar and/or butter, must also be considered for those products subjected to small batch processing, which would have a diluting effect on bioactives, including total (poly)phenols. Conversely further additional sources of (poly)phenols and antioxidants may be found in both apple butter—artisan and apple butter NPD, which included apple cider vinegar, reported to contain up to 290 mg L^–1^ (poly)phenols^46^, although this would only account for ~ 20 mg kg^–1^ in final butter NPD product. Spices such as cinnamon, also included, albeit in low quantities are known to contain high levels of flavonols^47,48^ as well as a range of antioxidants including cinnamaldehyde. This may partly explain an increase in antioxidant content on processing for these two products and ultimately the high antioxidant content in apple butter—artisan may not all be attributable to apple (poly)phenols. The addition of preservatives sodium metabisulphite and potassium sorbate to apple puree may also have influenced the retention of (poly)phenols in this product due to their own antioxidant properties^49,50^. Total (poly)phenol by assay and antioxidant content results were in good agreement with the literature for products prepared from whole apple^51–55^.

Ascorbic acid was affected by processing, with losses of up to 97.1% (diced apple) reported following the heat treatment and/or mechanical disruption of cells, where it is first oxidised by ascorbic acid oxidase to L-dehydroascorbic acid (DHAA), then to 2,3-diketogulonic acid, which has no biological activity^56^. Ascorbic acid can inhibit PPO by binding to the active centre of the enzyme, as well demonstrating strong reducing power by converting o-quinones produced by the action of PPO on (poly)phenols back to their phenol form and it is possible that these processes may also have reduced ascorbic acid content of products^57^. Indeed, a study on acidified Granny Smith apple puree reported initial losses on treatment in the range of 38.5–85.5%. Reductions observed for ascorbic acid for cooked products in our study is within the expected range^51^ excluding apple butter—artisan (55% reduction), which had high concentrations in raw material and may have more added.

Whilst there is currently no dietary reference intake for (poly)phenols in the UK, a suggestion of 500 mg day^–1^ has emerged based on the expected (poly)phenolic content of a diet achieving ‘5-a-day’ for fruit and vegetables^58^. A 100 g portion of apple sauce NPD would provide 146 mg of (poly)phenols, almost a third (29.2%) of this recommendation, whereas 10 g apple butter would deliver lesser quantities (apple butter—artisan, 7.3 mg and apple butter NPD, 14.3 mg). In the UK, nutritional data on food labels is described using a combination of reference intake and a front-of-pack traffic light system, where fat, sugar and salt are categorised as low (green), medium (amber) and high (red)^59,60^. While final product information is unknown for industrial intermediate products, they would be considered amber for sugar content at production. Apple sauce NPD was amber for sugar and fat content, while both apple butter products were in the red category for sugar content. While it may not be possible to prepare a concentrated apple product which would be low in sugar owing to the natural sugar content of apples, it is also important to consider the portion size of these products typically consumed when interpretating the overall sugar content of such products.

### Targeted investigation: pomace

Relative contributions of the different structural groups of (poly)phenols in pomace, confirm those previously reported by others^61,62^. Total (poly)phenol content however, was up to fourfold higher^18^, suggesting that pomace from the Armagh Bramley is a particularly good source of (poly)phenols. The higher concentrations observed in apple sauce NPD may be explained by differences in hand vs machine peeling. Dice produced 10–15% more pomace, and may have included more flesh with a lower (poly)phenol content than peel and core^63^. While ascorbic acid was present in apple pomace, the variance between samples (SD) was high, and this compound was quantified in only one of three batches for both dice and canned sliced apple. Ascorbic acid is not generally expected in apple pomace due to rapid oxidisation in the presence of oxygen^64^. Estimation of total (poly)phenol content by assay and antioxidant capacity by DPPH were also higher in this study than in previous studies^65^.

Although there were some efficient uses of apple pomace such as producing puree from dice pomace (2–4% overall), producers in NI often dispose of apple pomace as animal feed. Whilst pomace inclusion in feed is a viable supplement with beneficiary effects on lipid oxidation in meat observed, resulting in an extension of shelf life^66,67^, this potentially is not the most lucrative use of the high levels of (poly)phenolics uniquely present in Armagh Bramley pomace. Figures from 2014 demonstrated that there were 18.6 million people employed across Europe in the bio-economy of production of high-value by-products from food production, with 2.2 trillion Euros generated from waste streams in that year^68^. NI producers are on the exciting cusp of a potential circular economy with apple pomace (poly)phenols highly sought in a variety of industries including, food additives for preservation, polysaccharide nanoparticle applications, dietary supplements, cosmetics and pharmaceuticals^69–74^.

## Conclusion

The Armagh Bramley apple is a significant source of (poly)phenolic compounds as well as being high in antioxidant activity, which translates to relatively high levels of bioactives in its processed products. The processing conditions for the Armagh Bramley apple, however, significantly affect the (poly)phenolic profile and resulting content of the product. Products which have been subjected to industrial screw cooking contained the highest (poly)phenol content. Due to the high levels of phenolics in the Armagh Bramley, the resulting pomace by-products from processing is, so far, an untapped rich source of (poly)phenols with great potential for valorisation and added value to the economy.

## Supplementary Information

Supplementary Information.

## Data Availability

Data is provided within the manuscript or supplementary information files.
