# Supplementary material for: Effects of processing on the polyphenolic content of Armagh Bramley apple products and pomace
Source: Sci Rep. 2025 Dec 18;16:2035. doi: 10.1038/s41598-025-31695-7 (PMC12808679; doi:10.1038/s41598-025-31695-7)
Supplement: Supplementary file 1 — Supplementary Information. [file 41598_2025_31695_MOESM1_ESM.docx]

## Title: Effects of processing on the polyphenolic content of Armagh Bramley apple products and pomace

**Scientific Reports**

**Author names and affiliations:**

Ruth Loy*^a,b^, William C. McRoberts^a^, Alan Gordon^a^, Chris I.R. Gill^b^, L.K. Pourshahidi^b^.

a Agri-Environment Branch, Environment and Marine Sciences Division, Agri-Food and Biosciences Institute, Newforge Lane, Belfast BT9 5PX, UK

^b^ Nutrition Innovation Centre for Food & Health (NICHE), Ulster University, Coleraine, Northern Ireland, BT52 1SA, UK

**Corresponding author:**

Ruth Loy. Email: [Ruth.Loy@afbini.gov.uk](mailto:Ruth.Loy@afbini.gov.uk) ORCID: 0000-0001-6314-1933

**Description of production**

**Diced apple** - Following mechanical grading diced apple was prepared by firstly peeling and coring apples then employing Urschel dicing equipment to chop apple into 10mm^2^ dice. 40-45% of the starting material was discarded as pomace during processing.

**Apple puree** – Prepared from pomace which had been held in storage bins following apple dice production and subjected to screw cooking (100^o^C, 2-3minutes) before being passed through a sieve (0.8mm) to a holding hopper (Figure S1). The temperature of product was held at >90^o^C before being sieved again to hot fill 225kg plastic poly drums for dispatch. This process resulted in only 2-4% waste pomace remaining following the cooking process.

**Canned sliced apple** – Apples of <60mm or >110mm were discarded before washing, peeling, coring and slicing (Figure S2). Sliced apple was stored in a brine solution (1-2%) prior to blanching (88-95^o^C, 3minutes). Product was then canned and pasteurised (internal temperature of the cans increased to >71^o^C over 11.5 minutes). 30-35% of the starting material was discarded as pomace during this process.

**Industrial apple sauce, intermediate** – Apples were graded and prepared as described for canned sliced apple. Apples were poured into a screw crusher and subjected to screw cooking (95^o^C, 3minutes) before being passed through a sieve (2.0mm) as described in Figure S3. Product was then canned and pasteurized as described for canned sliced apple.

**Apple butter** - **artisan** – This award-winning product was prepared using apple puree prepared from Armagh Bramley apples, cider, brandy, treacle, sugar and spices. The producers declined to share the preparation process of the product but, typical values per 100g were as follows, fat 0.5g of which saturates <0.1g, carbohydrates 75.1g of which sugars 73.7g, fibre 2.3g, protein 0.8g and salt 0.3g

**Apple sauce, NPD** – Peeled, cored and chopped Armagh Bramley apple (2250g) were added to a pan with water (225ml) and simmered (8 minutes, 72±2^o^C). Butter (125g), (Kerrygold, Ornua foods, Staffordshire, UK LTD) and of caster sugar (250g), (Silver Spoon, Silver Spoon Company, Peterborough, UK) were then folded into the cooked apple.

**Apple butter, NPD** – Cored unpeeled apple (2730g), cider vinegar (300ml) (Aspall organic Cyder Vinegar, The Cyder House, Suffolk, UK), salt (2tsp) (Dri-Pak table salt, Dri-ak LTD, Ilkeston, UK) and water (480ml) were added to a pan and brought to the boil. Heat was reduced to allow the mixture to simmer (30 minutes, 72±2^o^C). The cooked apple was then pushed through a course sieve before the mix was returned to the original pan. Apple spice (2tbsp) of (Apple pie spice, McCormick & Co Inc, Maryland, USA) mix, vanilla extract (2tsp) (Tesco Madagascan Vanilla Extract, Tesco Ireland LTD., Co. Dublin, Ireland) and brown sugar (454g), (Billington’s Light Brown sugar, Silver Spoon, Peterborough, UK) were added and the was simmered (45min, 72±5^o^C).

1. BARRELS 2. SKINS/ APPLE 3. SODIUM 4. POTASSIUM 5. ACETIC

AND TANKS INTAKE METABISULPHITE SORBATE ACID *

INTAKE * INTAKE * INTAKE

**ALLERGEN SECURE**

**STORAGE**

6. BARREL 7. AUGER

AND TANK

*ADDITION ACCORDING TO CUSTOMER SPECIFICATION

WASH 9. DRY STORE

8. PUMP

10. HOPPER

SCREW

21. PACKAGING WASTE

11. COOKER (Temperature 100^o^C, 2-3min)

**PROCESS DELAY ACTION**

UNCOOKED SKINS ARE HELD IN STORAGE BINS UNTIL COOKING CAN RECOMMENCE. IF PROBLEM CANNOT BE SORTED BEFORE END OF WORKING DAY, ANY UNUSED MATERIAL IS TRANSFERRED TO WASTE TRAILER FOR ANIMAL FEED

22. SOLID MATERIAL I.E

12. SIEVE SKINS TRANSFERRED TO

WASTE TRAILER FOR ANIMAL

FEED

13. HOLDING

HOPPER

(Temp reached > 90°C)

14. PUMP AND SIEVE

15. HOLDING TANK

16. PRESERVATIVE

ADDITION

17. TANK/ BARREL FILLING

18. LABEL

19. STORE

20. DESPATCH

Figure S1. Industrial puree processing flow diagram as received from producer.

WATER APPLE RECEIPTS

APPLE STORAGE (CO2 7-8%)

GRADING REJECTS FOR ANIMAL FEED

PEELING/ SLICING SKIN REJECTS ANIMAL FEED/ SAUCE

WASH

WASHED VISUAL INSPECT/ TRIM REJECTS SAUCE

METAL DETECTION

PREPARATION ROOM

BRINE (1-2%) APPLE INTO BRINE

SALT FROM TRANSFER TO CANNING ROOM

STORE

APPLE TIPPED INTO

WATER TANK

DAMAGED CANS PALLETISED

WATER BAILED AND RECYCLED

HEATED BLANCHING (88-95^o^C, 3min)

INVERT CAN WEIGH CAN FILLING

CANNING ROOM

LIDS

LID & SEAL

CAN & LID STORAGE

SPRAY WASTE/WATER LICENSED

CONTRACTOR

CANS & LID RECEIPT COOK (can internal temp >71^o^C)

COOL

CHLORINATION OF TRANSFER TO LABEL ROOM

COOLING WATER DISINFECT CAN/ CONVERYER

STORE FOR 10DAYS

CHLORINE TABLET RECEPT INSPECT DAMAGES ANIMAL FEED

Figure S2. Canned sliced apple processing flow diagram as received from producer.

WATER APPLE RECEIPTS

APPLE STORAGE (CO2 7-8%)

GRADING REJECTS FOR JUICE

BLEMISHES/ CORE REJECTS ANIMAL FEED

VISUAL INSPECTION

METAL DETECTION

PREPARATION ROOM

BRINE (1-2%) STORED IN BRINE

SALT FROM TRANSFER TO CANNING ROOM

STORE

POURED INTO

SCREW CRUSHER

WATER SCREW COOKING (95^o^C, 3min, 2.0mm sieve)

HEATED PUMP TO FILLING STATION

INVERT CAN CAN WEIGH/ FILL

CANNING ROOM

LIDS LID & SEAL

CAN & LID STORAGE SPRAY

COOK (can internal temp >71^o^C)

COOL

CHLORINATION OF TRANSFER TO LABEL ROOM

COOLING WATER DISINFECT CAN/ CONVERYER

STORE FOR 10DAYS

CHLORINE TABLET RECEPT INSPECT DAMAGES ANIMAL FEED

Figure S3. Industrial apple sauce processing flow diagram as received from producer.

**QA data - (Poly)phenol analysis by compound**.

An initial recovery experiment was conducted to assess recovery of compounds measured in this study with procyanidin B1, procyanidin B2, catechin and epicatechin spiked at the equivalent of 280 mg kg^-1^ DW. Chlorogenic acid was spiked at the equivalent of 1000 mg kg^-1^ DW, quercetin 3-rutinoside, quercetin 3-glucoside, quercetin 3-rhamnoside and phloridzin spiked at the equivalent of 300 mg kg^-1^ DW. Finally, quercetin 3-galactoside was spiked at the equivalent of 321.8 mg kg^-1^ DW, with mean recovery across all compounds ranging from 89.8 to 116.0%. Following that a composite peel sample was prepared in lab and 10 replicates of this were analysed on 3 different occasions to prepare a reference material which was then analysed in duplicate alongside further sample batches. LOD and LOQ were derived by standard dilution series with signal to noise instrumentally monitored, LOQ was set at s/n 10:1 and LOD was set at s/n 3:1.

Table S1. Summary of QA data for (poly)phenol analysis by compound.

|  | Average validation recovery (n=5), % | | LOD s/n 3:1, LOQ s/n 10:1 mg kg^-1^ DW | | Average values for Peel reference material mg kg^-1^ DW | |  |
| --- | --- | --- | --- | --- | --- | --- | --- |
| (Poly)phenol | Flesh | Peel | LOD | LOQ | Study (n=22) | % RSD study | |
| Procyanidin B1 | 95.2 | 95.7 | 20 | 50 | 695 | 7.9 | |
| (+) Catechin | 104.2 | 98.1 | 20 | 50 | 280 | 11.2 | |
| chlorogenic acid | 105.1 | 96.5 | 10 | 20 | 1418 | 9.7 | |
| Procyanidin B2 | 100.7 | 110.6 | 20 | 50 | 2151 | 6.5 | |
| (-) Epicatechin | 104.8 | 116.0 | 20 | 50 | 1702 | 8.4 | |
| Coumaroyl quinic acid | N/A | N/A | 2 | 5 | 130 | 11.6 | |
| Quercetin 3-rutinoside | 90.3 | 92.8 | 2 | 5 | 59.8 | 10.0 | |
| Quercetin 3-galactoside | 89.8 | 101.8 | 2 | 5 | 418 | 10.2 | |
| Quercetin 3-glucoside | 96.2 | 95.5 | 2 | 5 | 167 | 9.5 | |
| Quercetin 3-rhamnoside | 91.5 | 97.8 | 2 | 5 | 315 | 7.9 | |
| Phloretin xyloglucoside | N/A | N/A | 1 | 2 | 481 | 10.5 | |
| Phloridzin | 92.8 | 97.8 | 1 | 2 | 645 | 6.8 | |
| Cyanidin 3-galactoside | N/A | N/A | 2 | 5 | N/A | N/A | |

* n= number of measurements

**QA data – Procyanidin content by phloroglucinolysis**

Phloroglucinolysis results in a mixture of flavan-3-ol monomers composed of existing monomers and terminal subunits along with phloroglucinol adducts which correspond to extension subunits. Due to the structure of the carbocation adduct it was not possible to prepare a spike to measure accuracy and an alternate method of validation was pursued. Composite flesh and peel samples were prepared and each extracted and analysed five times on three separate occasions to establish precision. Accuracy was then measured by analysing the same composite samples using an alternative method (Guyot et al., 2001) which used thiolysis and comparing results to the phloroglucinolysis method used in the study. The composite peel sample was then analysed in duplicate alongside further sample batches. LOD and LOQ were derived by standard dilution series with signal to noise monitored instrumentally, LOQ was set at s/n 10:1 and LOD was set at s/n 3:1.

Table S2. Summary of QA data for the analysis of proanthocyanidin content by phloroglucinolysis.

|  | LOD s/n 3:1, LOQ s/n 10:1 mg kg^-1^ DW | | Average values for Peel and flesh reference material LRM mg kg^-1^ DW | | | | | |
| --- | --- | --- | --- | --- | --- | --- | --- | --- |
|  | LOD | LOQ | Validation flesh (n=15) | Validation peel (n=15) | Comparison method Flesh (n=5) | Comparison method Peel (n=5) | Study (n=22) | %RSD study |
| Catechin | 36 | 180 | 1301 | 1382 | 1232 | 1333 | 1363 | 13.1 |
| Epicatechin | 36 | 180 | 1448 | 5328 | 1375 | 4818 | 5131 | 9.1 |
| Adduct | 36 | 180 | 5750 | 21421 | 5520 | 17825 | 21029 | 6.3 |
| Total | 36 | 180 | 8498 | 28131 | 8126 | 23975 | 27524 | 6.7 |

* n= number of measurements

GUYOT, S., MARNET, N. & DRILLEAU, J.-F. 2001. Thiolysis−HPLC Characterization of Apple Procyanidins Covering a Large Range of Polymerization States. *Journal of Agricultural and Food Chemistry,* 49**,** 14-20.

**QA data – Ascorbic acid content**

Initially a recovery experiment was conducted where a composite flesh sample was spiked at the equivalent of 1000 and 2000mg/kg DW on 5 samples on 3 separate occasions. Recovery ranged from 83.8 to 95.7%. A composite peel sample was prepared and extracted and analysed five times on three separate occasions which allowed the preparation of a reference material. LOD and LOQ were derived by standard dilution series with signal to noise monitored instrumentally, LOQ was set at s/n 10:1 and LOD was set at s/n 3:1 (Table S3). The peel reference material was then analysed in duplicate alongside further sample batches.

Table S3. Summary of QA data for the analysis of ascorbic acid

|  | Average validation recovery % (n=15) | | LOD s/n 3:1, LOQ s/n 10:1 mg kg^-1^ DW | | Average values for Peel and flesh reference material LRM mg kg^-1^ DW | | | |
| --- | --- | --- | --- | --- | --- | --- | --- | --- |
|  | Flesh low spike | Flesh high spike | LOD | LOQ | Validation flesh (n=15) | Validation peel (n=15) | Study Peel (n=14) | %RSD study |
| Ascorbic acid | 90.7 | 91.2 | 2 | 10 | 923 | 3040 | 3196 | 3.0 |

* n= number of measurements
